# Pleiotropic Locus 15q24.1 Reveals a Gender-Specific Association with Neovascular but Not Atrophic Age-Related Macular Degeneration (AMD)

**DOI:** 10.3390/cells9102257

**Published:** 2020-10-08

**Authors:** Christina Kiel, Tobias Strunz, International AMD Genomics Consortium (Project Manager Susan Blanton) IAMDGC, Felix Grassmann, Bernhard H. F. Weber

**Affiliations:** 1 Institute of Human Genetics, University of Regensburg, 93053 Regensburg, Germany; Christina.Kiel@klinik.uni-regensburg.de (C.K.); Tobias.Strunz@klinik.uni-regensburg.de (T.S.); felix.grassmann@abdn.ac.uk (F.G.); 2 John P. Hussman Institute for Human Genomics, University of Miami, Miami, FL 33136, USA; sblanton@med.miami.edu; 3 Institute of Medical Sciences, University of Aberdeen, King’s College, Aberdeen AB24 3FX, UK; 4 Institute of Clinical Human Genetics, University Hospital Regensburg, 93053 Regensburg, Germany

**Keywords:** age-related macular degeneration, choroidal neovascularization, pleiotropy, miRNA variant, eQTL, locus analysis, gender specificity

## Abstract

Genome-wide association studies (GWAS) have identified an abundance of genetic loci associated with complex traits and diseases. In contrast, in-depth characterization of an individual genetic signal is rarely available. Here, we focus on the genetic variant rs2168518 in 15q24.1 previously associated with age-related macular degeneration (AMD), but only with suggestive evidence. In a two-step procedure, we initially conducted a series of association analyses to further delineate the association of rs2168518 with AMD but also with other complex phenotypes by using large independent datasets from the International AMD Genomics Consortium (IAMDGC) and the UK Biobank. We then performed a functional annotation with reference to gene expression regulation based on data from the Genotype-Tissue Expression (GTEx) project and RegulomeDB. Association analysis revealed a gender-specific association with male AMD patients and an association predominantly with choroidal neovascularization. Further, the AMD association colocalizes with an association signal of several blood pressure-related phenotypes and with the gene expression regulation of *CYP1A1*, a member of the cytochrome P450 superfamily of monooxygenases. Functional annotation revealed altered transcription factor (TF) binding sites for gender-specific TFs, including SOX9 and SRY. In conclusion, the pleiotropic 15q24.1 association signal suggests a shared mechanism between blood pressure regulation and choroidal neovascularization with a potential involvement of CYP1A1.

## 1. Introduction

Over the past 15 years, genome-wide association studies (GWAS) have revolutionized research into complex diseases and have led to an increasingly comprehensive knowledge about their genetic fundamentals. While the first successful GWAS was on age-related macular degeneration (AMD), describing a reproducible locus on the long arm of chromosome 1 [1], the most recent AMD GWAS reported 34 loci harboring 52 independent genetic lead signals with genome-wide significance [2]. Despite having generated a refined map of loci contributing to AMD pathology, follow-up studies, which investigate the biological mechanisms underlying the statistical associations, are still greatly missing. Not surprisingly, AMD-related research mainly focuses on *CFH* and *ARMS2*/*HTRA1*, two loci, which display the largest effect sizes for AMD risk [2,3].

While GWAS approaches follow an unbiased strategy, targeted association studies have the advantage to provide an improved statistical power while focusing on genetic variants or genes for which further information on possible disease-related aspects are available. To this end, one category of promising genes are microRNAs (miRNAs), which are considered excellent disease biomarkers [4] and even encouraging therapeutic targets [5,6], specifically for complex diseases. MiRNAs are small post-transcriptional regulators by incorporating into the RNA-induced silencing complex (RISC), which binds to mRNA transcripts in the cytosol and contributes to their degradation or translational repression [7]. Of special interest is the seed region of the mature miRNA, a key feature for target gene recognition and binding [8,9]. Genetic variants located in miRNAs could therefore be of functional importance as these may influence gene expression regulation and thus contribute to a pathological phenotype. Using a targeted association analysis for miRNA variants, Ghanbari and colleagues (2017) identified an association of AMD with the genetic variant rs2168518, which is located in the seed region of hsa-mir-4513 [10]. A recent meta-analysis of AMD further supported the association of this variant with AMD [11].

Interestingly, the miRNA seed variant rs2168518 seems to have a pleiotropic effect as it has been linked to several complex traits and diseases, including cardiometabolic phenotypes, the clinical outcome in coronary artery disease and prognosis of lung adenocarcinoma [12,13,14,15]. However, so far, the potential mechanism behind these associations has always been limited to the obvious candidate, the hsa-mir-4513 gene itself. Here, we aimed to analyze in-depth the AMD-associated 15q24.1 interval harboring the rs2168518 signal. We have performed a series of analyses to investigate the association with AMD and to elucidate its pleiotropic nature. Furthermore, we have carried out several analyses to functionally characterize this locus with regard to gene expression regulation and transcription factor (TF) binding.

## 2. Materials and Methods

### 2.1. Availability of Datasets

The dataset from the Resource for Genetic Epidemiology Research on Adult Health and Aging (GERA) cohort, a sub-study of the Research Program on Genes, Environment, and Health (RPGEH), is available at the database of genotypes and phenotypes (dbGap) under the accession number phs000674.v3.p3 and phs000788.v2.p3, respectively. The data permitted for sharing by the respective institutional review boards from the International AMD Genomics Consortium (IAMDGC) is available at dbGap under the accession number phs001039.v1.p1. The dataset from the Genotype-Tissue Expression Project (GTEx) is available at dbGap under the accession number phs000424.v8.p2. Processed gene expression data of GTEx can be downloaded at www.gtexportal.org. The UK Biobank dataset can be obtained upon application (ukbiobank.ac.uk). This research has been conducted using the UK Biobank Resource under application number 44862. GWAS summary statistics of the UK Biobank data are publicly available at http://www.nealelab.is/uk-biobank/.

### 2.2. Description of Datasets

In total, data from 63,156 individuals, 19,018 AMD patients and 44,138 control individuals, were included to investigate the association of rs2168518 with AMD. To this end, data from the GERA cohort and the IAMDGC were jointly analyzed. The GERA cohort includes 2874 AMD patients and 26,306 control individuals only from European descent and born before 1948. The GERA dataset comprises 12,734 males and 16,446 females. Detailed information on array design [16], genotyping process and quality control (QC) [17], and imputation [18] is available. Further, data from the IAMDGC with 16,144 late-stage AMD patients and 17,832 control individuals were used. Unrelated individuals of the IAMDGC study were of European descent and included 14,352 males and 19,624 females. Subphenotypes within the late-stage AMD samples were 10,749 choroidal neovascularization AMD (CNV), 3235 geographic atrophy AMD (GA), and 2160 patients with CNV and GA combined. The IAMDGC data additionally contain 6657 individuals with early or intermediate AMD, which were included in the early AMD association analysis. Detailed information about gender distribution in the different subgroups are available in Supplementary Table S1. Detailed information about selection criteria, ophthalmological grading, QC of the genetic data, and imputation procedures were described in detail elsewhere [2].

To investigate the pleiotropic nature of rs2168518, we explored its association with multiple phenotypes as given in the UK Biobank dataset with publicly available GWAS summary statistics of the 2419 phenotypes available [19]. Summary statistics were evaluated for both sexes for a total of 361,194 individuals, and separately for females (*n* = 194,174) and males (*n* = 167,020). Further, we directly used UK Biobank data to extract specific phenotype information. In our analysis, only self-reported white individuals were included, and related individuals up to the third degree of kinship were excluded, as well as individuals with inconsistencies in self-reported sex and genetic sex. In total, 379,356 individual raw datasets remained in the study, including 204,527 females and 174,829 males.

Calculation of expression quantitative trait loci (eQTL) was performed in the GTEx data version 8. The detailed data processing protocols for the GTEx project are described elsewhere [20]. A genotype principle component analysis based on 100,000 randomly selected genetic variants from the GTEx whole genome sequencing data and the corresponding genotype information of the 1000 Genomes Project reference panel (Phase 3, release 20130502) [21] was conducted using the snpgdsPCA package [22] in R [23]. The first two principal components were plotted to determine the ethnicity. Only samples clustering next to the European reference individuals remained in the study, leaving 694 individuals (237 females and 457 males) for eQTL analysis. Sample sizes for the 49 analyzed tissues varied between 65 (see “kidney cortex”) and 584 (see “muscle skeletal”).

### 2.3. Association of rs2168518 with AMD

To investigate the association of rs2168518 with AMD, a combined analysis of the IAMDGC and GERA data was performed. Prior to analysis, genetic information of rs2168518 was extracted from the IAMDGC dataset. In the GERA dataset, genetic information of rs1378942, a proxy in full linkage disequilibrium (LD) with rs2168518, was extracted. Genetic information was converted to dosages. To calculate the association with AMD in the GERA and IAMDGC dataset, a logistic regression model was applied by using the *glm* function implemented in R [23], with adjustments for dataset and gender. The *p*-value threshold was set to 5 × 10^−8^ to identify genome-wide significant associations in the combined analysis.

Association analysis with different subgroups of AMD was performed exclusively in the IAMDGC dataset. A logistic regression model was applied as described above. The list of co-variates included age, gender, the first two genotype principal components, as well as the source of DNA. *P*-values were adjusted for multiple testing according to the false discovery rate (FDR, Q-value) [24] as implemented in the multtest package [25].

For the conditional analysis, a logistic regression model was used including all late-stage AMD samples of the IAMDGC dataset and adjusted for all covariates from the subgroup analysis. The analysis was further adjusted for the genetic variant with the smallest *p*-value of AMD association in the region of interest (rs11072508, R^2^ to rs2168518 = 0.903 in Europeans).

### 2.4. Haplotype Distribution over Different Populations

To investigate the allele frequency distribution of rs2168518 in different populations, all genetic variants in LD with rs2168518 (defined as R^2^ > 0.8 in Europeans) were extracted via the LDproxy tool from LDlink [26,27] (Supplementary Table S2). In total, 26 genetic variants were identified and used to investigate haplotype differences in various populations using the LDhap tool from LDlink. One variant (rs36117428) was excluded from the haplotype analysis due to missing data in the reference panel.

### 2.5. Phenome-Wide Association Analysis of rs2168518 in the UK Biobank Database

To investigate the association of rs2168518 with defined phenotype we used the preliminary UK Biobank PheWeb, which was based on the first Neale lab analysis available at the PheWeb browser [28]. PheWeb compromises GWAS results of 2419 phenotypes and is based on data of ~337,000 unrelated British individuals from the UK Biobank dataset [29]. All associations of rs2168518 below the *p*-value threshold of 10^−4^ were extracted. This filter resulted in 15 phenotype associations with rs2168518. Further, the association of rs2168518 with trait “Eye problems/disorders: Macular degeneration” was retested as it was not among the initial list of 15 phenotypes identified. GWAS summary statistics of UK Biobank data for the 15 significant phenotypes, and for “Eye problems/disorders: Macular degeneration”, were downloaded from http://www.nealelab.is/uk-biobank/ [19]. We retrieved summary statistics for GWAS from both sexes, as well as for female and male separately. Locuszoom plots [30,31] were generated to visualize the GWAS summary statistics. These plots were used to manually narrow the region of interest for all 16 phenotype associations to a region of 0.6 Mbp (Chromosome 15: 75,000,000–75,600,000, GRCh37). It should be noted that this cutoff was chosen to ensure inclusion of all association signals of the phenotypes included in the study.

### 2.6. Colocalization Analysis Based on the UK Biobank GWAS Summary Statistics

We performed colocalization analyses to test for gender specificity based on the UK Biobank summary statistics. This analysis was performed using the coloc package [32] in R [23] by comparing GWAS results from females and males of the same phenotype in the refined genomic region. Coloc probabilities indicate whether the association signal is found for one trait only, and not for others (in our case with male or female individuals). Probability thresholds of > 0.8 were applied for filtering the results.

### 2.7. Colocalization Analysis of the Genetic Signals Underlying UK Biobank Associations and AMD

A colocalization analysis using coloc was performed to test for correlation of AMD association signals in the IAMDGC dataset with phenotypes from the UK Biobank dataset. To this end, coloc requires association results, information about the proportion of cases for continuous phenotypes and the phenotype standard deviation for quantitative traits. For continuous traits at UK Biobank, the number of missing samples in the GWAS summary statistics for the respective phenotype were reported exclusively with sexes merged. To estimate the number of missing samples for females and males separately, we utilized the total sample size of the respective gender-specific GWAS and deducted the number of missing individuals of the respective phenotype divided by 2 with the assumption, that the missing individuals were spread evenly across the two sexes. Standard deviations for quantitative traits were retrieved either from the initial assessment visit of the UK Biobank Data Showcase [33] (for “Creatinine (enzymatic) in urine” and “Sodium in urine”) or directly from UK Biobank data concerning diastolic blood pressure (automated reading, DBP) and systolic blood pressure (automated reading, SBP). The latter two traits were determined for both sexes and separately for females and males.

Coloc probabilities indicate whether there is an association in the respective genomic region for both traits. In this study, we used probability thresholds of > 0.8.

### 2.8. eQTL Calculation

eQTL calculations were performed for all 49 tissues provided by GTEx (version 8) [20]. Phased genotypes were converted into allele dosages and a minor allele frequency threshold of 1% was applied. Local eQTL were calculated based on linear regression models using FastQTL (version v2.184_gtex) [34] and an arbitrary window of 1 Mbp up- and downstream of the variant or gene of interest was selected.

We first identified genes potentially regulated by rs2168518 using an exploratory *p*-value < 10^−4^. Thereafter, we calculated all local eQTL for the significant rs2168518 eQTL genes (eGenes). These results were then used for colocalization analyses regarding other eQTL or GWAS signals. Further, by miRWalk 3 [35,36], we examined whether identified eGenes are predicted target genes of hsa-mir-4513.

### 2.9. Identification of Transcription Factor Binding Sites

All genetic variants in LD with rs2168518 (defined as R^2^ > 0.8 in Europeans, Supplementary Table S2) were analyzed regarding their impact on TF binding by RegulomeDB 2.0 [37,38]. Variants with a RegulomeDB rank < 3 were selected for further investigations.

## 3. Results

### 3.1. Association of rs2168518 with AMD

#### 3.1.1. Validation of a Genome-Wide Significant Association of rs2168518 with AMD

**Figure 1:**
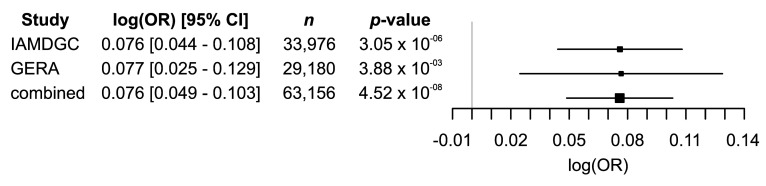
Genome-wide association of age-related macular degeneration (AMD) with rs2168518. The association was calculated separately in the International AMD Genomics Consortium (IAMDGC) and Genetic Epidemiology Research on Adult Health and Aging (GERA) datasets, as well as in the combined data where the association of AMD with rs2168518 displays genome-wide significance (*p*-value < 5 × 10^−8^). For all associations, the A allele indicates the effect allele. OR = odds ratio, CI = confidence intervals, *n* = sample size.

A recent meta-analysis considering three datasets reported a genome-wide significant association of AMD with rs1378940, a proxy variant of rs2168518 (R^2^ = 1 in European individuals) [11]. However, this study did not follow a classical GWAS approach, but instead relied on a multivariate GWAS model introduced as multiple trait analysis of GWAS (MTAG) [39]. For this, only summary statistics were used and more critical datasets that are not entirely independent. We therefore wanted to validate the association of rs2168518 with AMD in the original and independent data from the IAMDGC and GERA cohorts. In both datasets, a nominal association with AMD was observed (*p*-value IAMDGC = 3.05 × 10^−6^ and GERA = 3.88 × 10^−3^). Combining the two datasets provided the statistical power to reach genome-wide significance of an association of AMD with rs2168518 (*p*-value = 4.52 × 10^−8^) (Figure 1).

#### 3.1.2. Refinement of the Genetic Association Signal with AMD

To further characterize the potential influence of rs2168518 on AMD, we calculated its association with AMD subtypes including late-stage AMD, GA, CNV, GA and CNV combined, as well as early stage AMD (Table 1).

**Figure 2:**
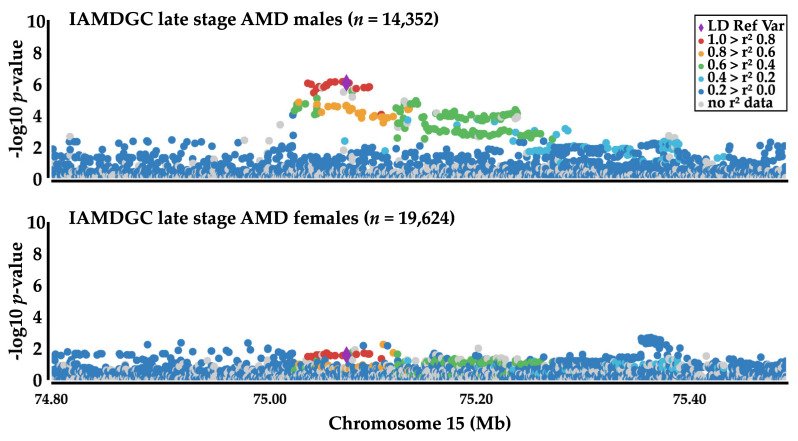
Association of rs2168518 with AMD in males and females. The association with late-stage AMD was calculated separately for both sexes in the IAMDGC dataset. While an association signal with AMD can be detected in males, an association in females is missing. Rs2168518 is presented as purple diamond and serves as linkage disequilibrium (LD) reference variant. Plots were created with Locuszoom [30]. *n* = sample size.

This revealed an association of rs2168518 with late-stage AMD, GA, and CNV (Q-value < 0.05). No association was detected between rs2168518 and the combined GA and CNV phenotype. However, the number of individuals in this subgroup was relatively small with 2160 cases and therefore statistical power might be insufficient. Comparison of the two other late-stage AMD subtypes revealed, that the association with GA (Q-value = 0.024) was weak in comparison to the association with CNV (Q-value = 1.87 × 10^−5^). Further, we separated the IAMDGC dataset into individuals younger and older than 75 years of age. Interestingly, the association of rs2168518 with AMD was evident in the group of individuals under the age of 75 (Q-value = 1.87 × 10^−5^). Dividing the IAMDGC dataset according to gender revealed rs2168518 to be strongly associated with AMD in males (Q-value = 4.00 × 10^−6^), but only weak with AMD in females (Q-value = 0.038) (Figure 2).

This finding is strengthened by the fact, that the female sample sizes were larger (*n* AMD cases = 9612, *n* controls = 10,012) in comparison to the male sample sizes (*n* AMD cases = 6532, *n* control = 7820). Taken together, rs2168518 reveals a gender-specific CNV-related association with AMD, most strongly when under the age of 75 years.

A conditional analysis was done to clarify whether the AMD association signal at the 15q24.1 locus consists of one or more genetic signals. As a result, no further variants were significantly associated with AMD, suggesting that the association with AMD at this locus was conferred exclusively by a single signal (Supplementary Figure S1).

### 3.2. Haplotype Distribution in Populations

Next, we tested whether rs2168518 exhibits a population-specific effect. We defined haplotypes based on all genetic variants in LD with rs2168518 (R^2^ > 0.8 in Europeans, Supplementary Table S2). This resulted in seven haplotypes, H1 to H7 (Supplementary Table S3). Interestingly, haplotype frequencies varied widely between populations (Table 2).

The haplotype H1, which contains the rs2168518 AMD risk allele “A”, showed by far the highest allele frequency in Europeans (55.77%). In contrast, H2 is the most frequent haplotype in other populations. Our results show that only a minority of the Asian and African populations carry the rs2168518 AMD risk allele.

### 3.3. Pleiotropic Effect of rs2168518 Assessed in the UK Biobank Data

To investigate a pleiotropic effect of rs2168518, we used the PheWeb browser [28] containing GWAS results of over 2000 phenotypes in the UK Biobank dataset. We applied an exploratory threshold for significance (*p*-value < 10^−4^) and detected significant associations of rs2168518 with 15 phenotypes, of which eight reach genome-wide significance (*p*-value < 5 × 10^−8^) (Table 3, Supplementary Table S4).

We manually catalogued the significant phenotypes into related groups and investigated gender-specificity and colocalization with the IAMDGC AMD signal.

The UK Biobank phenotype code for AMD “Eye problems/disorders: Macular degeneration” failed to reach the set *p*-value threshold, which may be attributed to the low sample size of AMD patients in the UK Biobank cohort. The association analysis was performed with almost 109,000 samples stated by the PheWeb browser [28], while the actual version of UK Biobank reports approximately 5000 self-reported AMD cases according to data field 6148 in the UK Biobank Data Showcase [33].

#### 3.3.1. Association of rs2168518 with Blood Pressure Phenotypes

The majority of significant phenotypes, 10 out of 15, were assigned to one of the blood pressure-related groups (Table 3). The AMD risk increasing “A” allele of rs2168518 displays a consistent protective effect on high blood pressure measurements, including DBP, SBP, and hypertension. This was supported by significant associations with the same effect direction of indirect hypertension measurements including the treatment with Ramipril (“Treatment/medication code: ramipril”) and bendroflumethiazide (“Treatment/medication code: bendroflumethiazide”). In addition, GWAS with focus on control individuals in these groups (“Vascular/heart problems diagnosed by doctor: None of the above”, “Medication for cholesterol, blood pressure or diabetes: None of the above”) displayed adverse effect sizes for the same allele.

Interestingly, only the association signal of Ramipril treatment displays a gender-specific association signal in this group with rs2168518 being associated with Ramipril treatment exclusively in males (*p*-value = 4.03 × 10^−8^), while there is no association of rs2168518 with Ramipril-treated females (*p*-value = 0.103).

**Figure 3:**
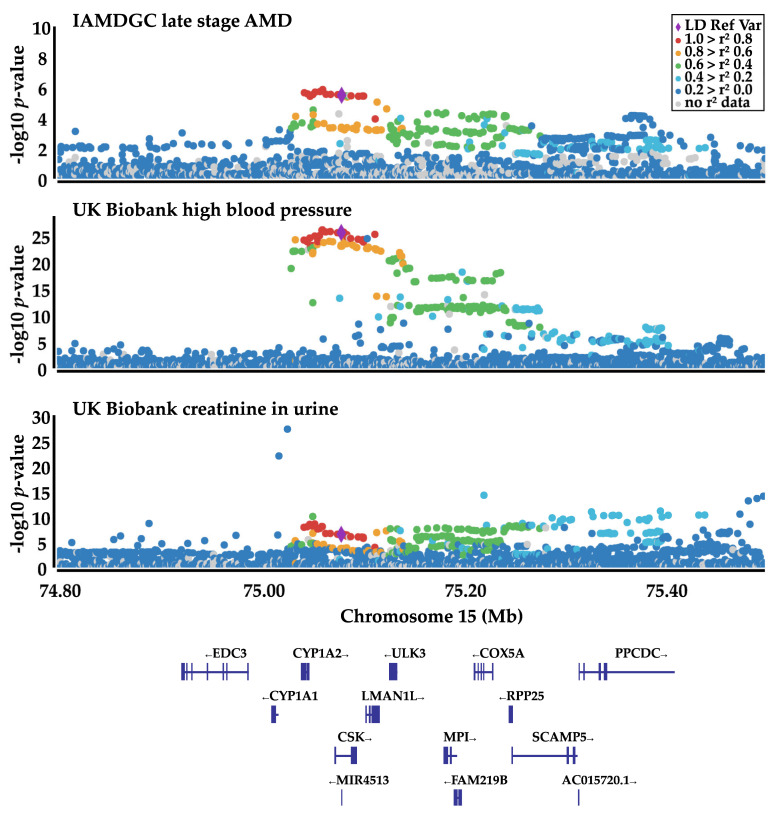
Locuszoom plots of the association signals in late-stage AMD (IAMDGC), “Vascular/heart problems diagnosed by doctor: High blood pressure” (UK Biobank) and “Creatinine in urine” (UK Biobank). The genetic association signal of AMD represents the same genetic signal as for the blood pressure association (coloc probability = 0.976), while the “UKB creatinine in urine” association underlies a different genetic signal (coloc probability = 0.037). Rs2168518 is presented as a purple diamond and serves as LD reference variant. Plots were created with Locuszoom [30].

The colocalization analysis with AMD shows that the association signal underlying the blood pressure GWAS is identical to the AMD association in the IAMDGC cohort (coloc probabilities for the same signal > 0.8, Figure 3).

This is the case for most of the phenotypes in all sub-studies (both sexes, as well as female and male) in comparison with AMD (both sexes and male). Exceptions are DBP and SBP, which only display a coloc probability > 0.8 with AMD in females.

#### 3.3.2. Association of rs2168518 with Metabolic Products in Urine

A second group of rs2168518-associated traits can be formed by metabolic products in urine, creatinine (“Creatinine (enzymatic) in urine”), and sodium (“Sodium in urine”). The “A” allele of rs2168518 showed a protective association with both phenotypes against high creatinine/sodium levels in the urine. There was no difference in the association signal between females and males. Interestingly, the colocalization analysis with AMD revealed that the association signal for both phenotypes was a different genetic signal than for AMD (coloc probability for different genetic signals > 0.8, Figure 3).

#### 3.3.3. Association of rs2168518 with Other Phenotypes

In the UK Biobank cohort, three further phenotypes were significantly associated with rs2168518 (*p*-value < 10^−4^). Strikingly, the phenotype “Hearing difficulty/problems with background noise” showed a gender-specific difference in its association with rs2168518 opposite to AMD with a strong association in females (*p*-value = 6.01 × 10^−9^) and no association in males (*p*-value = 0.345). The colocalization analysis in females and males further supports this finding (probability for a signal in females only = 0.886). The genetic association signal for the UK Biobank phenotypes “Hearing difficulty/problems with background noise” (both sexes and females only) and “Mineral and other dietary supplements: Glucosamine” (both sexes) both colocalized with the IAMDGC AMD signal (both sexes and males only).

### 3.4. Local eQTL Analysis of rs2168518 in the GTEx Dataset

Next, we investigated whether rs2168518 has an impact on gene expression regulation. A local eQTL analysis was conducted in 49 tissues as given in the GTEx project. An exploratory *p*-value threshold of 10^−4^ resulted in 93 eQTL of rs2168518 and 14 eGenes across all tissues (Supplementary Table S5). Seven of the eGenes displayed a rather tissue-specific eQTL in less than five tissues and showed mostly moderate *p*-values (*p*-value > 10^−7^ for 5 out of 7 eGenes). In contrast, the smallest *p*-value with 3.78 × 10^−33^ displays *RPP25* (see “artery tibial”), which is a significant eGene in 17 tissues.

We further calculated eQTL by separating male and female data and compared their association signals by a colocalization analysis. A preliminary interpretation of these results failed to show a gender-specific effect on gene expression regulation. An interpretation of these results should be made with caution due to an uneven gender distribution in the GTEx dataset (237 females vs. 457 males).

#### 3.4.1. eGenes Are Not Regulated by hsa-mir-4513

To investigate the probability that eGenes are regulated by hsa-mir-4513, we used miRWalk 3 [35,36] to predicted target genes of the respective miRNA. Some eGenes exhibit potential hsa-mir-4513 binding sites, but none of these genes were a validated target defined by miRTarBase [40]. The prediction tools TargetScan [41] and miRDB [42] did not suggest the eGenes as targets of hsa-mir-4513 (Supplementary Table S6). Therefore, it appears unlikely that hsa-mir-4513 was responsible for the observed effects on gene expression regulation in this region.

#### 3.4.2. Colocalization of eGenes

Beside a classical eQTL analysis, a colocalization analysis of eGenes was performed to investigate whether the respective eGenes were regulated by the same genetic signal. This analysis was performed for all tissues separately that display at least two local eQTL regarding rs2168518. We observed three gene pairs, which were regulated by the same genetic signal in at least two tissues (coloc probability > 0.8) (Supplementary Table S7). The gene pair that occurred most frequently was *ULK3* and *CSK* in four tissues, followed by *RPP25* and *SCAMP5* in three tissues, and *SCAMP2* and *MPI* in two tissues. An exemplary colocalization heatmap is shown in Supplementary Figure S2 for adipose subcutaneous tissue, which displays most eQTL results.

#### 3.4.3. Colocalization with Phenotypes

**Figure 4:**
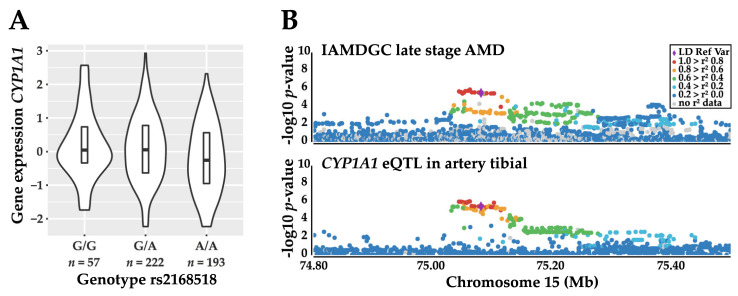
Expression quantitative trait locus (eQTL) analysis reveals *CYP1A1* as a potentially regulated gene by the AMD-associated variant rs2168518. (**A**) As an example, the significant eQTL gene (eGene) *CYP1A1* is differentially regulated by rs2168518 in artery tibial tissue and (**B**) colocalizes with the genetic association signal of AMD. Manhattan Plots were created with Locuszoom [30].

To investigate whether an eQTL signal corresponds to the same genetic signal as a phenotype association, a colocalization analysis of eQTL with the IAMDGC data and UK Biobank summary statistics was performed. Overall, the signals of five eGenes correspond to the genetic signals, which were also associated with AMD (Supplementary Table S8). Of these five eGenes, only one, namely *CYP1A1* (Figure 4), revealed a colocalization in more than two tissues.

Furthermore, colocalization of *CYP1A1* occured frequently with ten different blood pressure phenotypes. However, for some of these phenotypes, this signal was not as sharply separated from the other eGenes like for AMD. In these cases, *CYP1A1* colocalized with the genetic association of the respective phenotypes in combination with *ULK3* and *CSK*.

In contrast to AMD and the blood pressure phenotypes, the association signal of “Birth weight of the first child” colocalized clearly with *RPP25* in several tissues. Whereas, the association signal of “Creatinine in urine” and “Sodium in urine” failed to colocalize with any eQTL.

### 3.5. Functional Annotation of Genetic Variants at 15q24.1 with RegulomeDB 2.0

**Figure 5:**
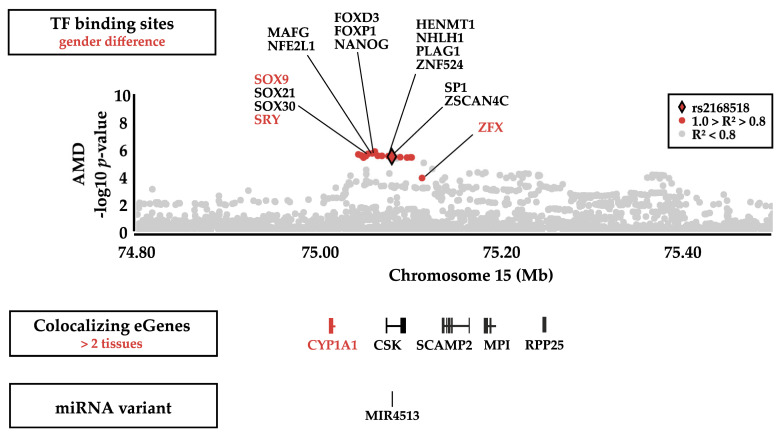
Locus scheme of functional mechanisms within the AMD association signal. The Manhattan plot represents the association *p*-values with late-stage AMD. rs2168518 is highlighted as a red diamond and all variants strongly linked with rs2168518 (R^2^ > 0.8 in Europeans) are displayed as red dots. Potentially altered transcription factor (TF) binding sites are shown with regard to the approximate chromosomal position. TFs related to gender differences, due to their location on sex chromosomes (SRY and ZFX) or because they are direct target genes of TFs located on sex chromosomes (SOX9), are highlighted in red. Below the Manhattan Plot are schemes of eGenes colocalizing with the AMD association signal and eGenes are displayed in regard to their approximate chromosomal position. eGenes colocalizing with the AMD association signal in more than two tissues are highlighted in red. As rs2168518 is located within hsa-mir-4513, the chromosomal position of this microRNA (miRNA) is shown below the colocalizing eGenes with AMD. The raw version of the Manhattan Plot was created with Locuszoom [30].

Variants in TF binding sites allow to address potential transcriptional regulation mechanisms. We investigated whether genetic variants in LD with rs2168518 (R^2^ > 0.8 in Europeans, Supplementary Table S2) have an impact on TF binding by Regulome DB 2.0 [37,38]. In total, eight of 27 variants were ranked from Regulome DB to have a potential influence on TF binding or gene expression regulation (RegulomeDB rank < 3, Supplementary Table S9). Two of them were predicted to have a regulatory impact on *ULK3* expression in monocytes, but showed little evidence to match TF motifs. The remaining six variants were predicted to impact TF binding by matching DNase peaks and footprints. Interestingly, one of these variants (rs11072507) altered a motif targeted by SRY and SOX9, which play an important role in sex determination [43], and another variant (rs11636952) altered a motif targeted by ZFX, a TF located on the X chromosome (Figure 5).

## 4. Discussion

Here, we report on an in-depth analysis of a recently described AMD-associated locus at 15q24.1 [10,11]. This locus gained a wider interest as it harbors a genetic variant, rs2168518, speculated to modify hsa-mir-4513 function [10]. We first confirmed an earlier association of this locus with AMD in a large independent dataset and determined genome-wide significance for rs2168518 with AMD. We noticed a strong association of rs2168518 with the CNV complication in late-stage AMD and, additionally, a marked gender-specificity for male AMD patients. Further, the rs2168518 risk allele was found to be abundantly present in European individuals, but to a much lesser extent in an Asian or African background. Searching for pleiotropic effects of rs2168518, we demonstrated that colocalization of this genetic marker with GWAS summary statistics from over 2000 phenotypes at UK Biobank points to signal overlaps specifically with high blood pressure phenotypes. This is in line with a prior study [12], thus further supporting the importance of this locus for CNV genetics. We then proceeded to functionally annotate the 15q24.1 locus. An eQTL analysis in the GTEx database indicates that rs2168518 influences gene expression of 14 genes, seven of which with a measurable effect in five or more tissues. One of the eGenes stands out based on its colocalization with AMD and blood pressure association signals, namely *CYP1A1*. Notably, prediction tools to identify miRNA target genes failed to suggest *CYP1A1* as a target of hsa-mir-4513. This makes an indirect regulatory effect of *CYP1A1* through this miRNA unlikely. Finally, investigating TF binding provides a first clue to a possible mechanism explaining the gender-specificity of the rs2168518 association with AMD by suggesting an altered binding of gender-specific TFs.

Genetic correlations of complex diseases are a well-known phenomenon [44,45], which have the potential to uncover shared mechanistic processes between seemingly unrelated complex diseases. Also, an association of rs2168518 with several phenotypes has been described before, including cardiometabolic traits and the prognosis of lung adenocarcinoma [12,13,14,15]. Therefore, we were interested to further investigate this potential pleiotropic effect of rs2168518. An initial test with UK Biobank data revealed association of rs2168518 with several phenotypes, including blood pressure-related phenotypes, hearing difficulty/problems with background noise, birth weight of the first child, and metabolites in urine. Remarkably, a colocalization analysis revealed that only the blood pressure-related phenotypes as well as hearing difficulty/problems with background noise association signals correspond to the identical genetic signal as established for the AMD association. In contrast, the association signal for metabolites in urine clearly failed to match the same signal. This highlights the importance to consider the full spread of association signals at a defined locus instead of simply analyzing single variant associations.

The genetics of AMD and blood pressure and the correlation between these two traits were investigated by several studies [46,47,48]. In our own work, we established a combined protective effect of hypertension-associated variants in AMD [48], which is in line with the results of the current study. In contrast, phenotype association studies of AMD and hypertension pointed in the opposite direction [46,47]. The latter two association studies suggested that there may be a difference in the association between hypertension and CNV versus non-neovascular AMD. This in turn would indicate different underlying pathomechanisms that are more similar to high blood pressure in CNV than in non-neovascular AMD [46,47]. Such a hypothesis would fit our observations, namely that the investigated locus is mainly associated with blood pressure-related phenotypes and CNV, and to a lesser extent with other subtypes of AMD.

Often, genetic association studies simply focus on the discovery of association signals, while deeper analyses into underlying mechanisms are regularly sparse. Nevertheless, it is exactly the insight into such mechanisms that have the potential to increase our understanding of disease pathology [49]. Therefore, we focused on potential functional consequences of the identified association signals. We reported several genes for which expression is regulated by rs2168518 and we even revealed small genetic sub-signals that regulate multiple eGenes. The linkage of eGenes with phenotype association signals at the 15q24.1 locus reveals three main findings: First, the association signals of AMD and the blood pressure-related phenotypes colocalize to a great extent with the expression regulation of *CYP1A1,* for example, in the tibial artery and subcutaneous and visceral omentum adipose tissue. Since *CYP1A1* is not completely independent in its regulation of *CSK* and *ULK3*, these genes also show a colocalization in some tissues. Interestingly, *CYP1A1* appears not to be expressed in the human retina as reported by three recent RNA-sequencing studies [50,51,52]. It should be mentioned that the retina is a highly specialized tissue consisting of a variety of cell types, including at least 60 distinct types of neurons [53]. Drawing conclusions from current single cell RNA-sequencing studies of the retina is still difficult due to the low sample sizes and high inter-sample variability [51,54,55,56]. Further, the transcriptomes of the retinal supporting system, including retinal pigment epithelial cells and choroidal vasculature, are not well investigated on the transcriptome level so far [51,57]. The second main finding of the linkage analysis of eGenes with phenotype association signals at 15q24.1 revealed that the expression regulation of *RPP25* colocalizes with the association signal of “Birth weight of first child” in several tissues. Therefore, this finding appears to have a different molecular mechanism than the other phenotypes with an association signal at this locus. Third, there is no gene for which expression regulation colocalizes with the GWAS associations in the studies “Creatinine in urine” and “Sodium in urine”.

CYP1A1, a member of the cytochrome p450 enzyme family, has an important role in the detoxification reaction of polycyclic aromatic hydrocarbons, found, for example, in tobacco smoke and hence is involved in oxidative stress responses [58,59]. Oxidative stress has been noted as an important player in AMD pathogenesis [60]. In the pre-GWAS era, this had led to directly investigate associations between genes involved in oxidative stress defenses and AMD [61]; however, so far, AMD has not been linked to *CYP1A1*. Interestingly, in an animal model for AMD retinal *CYP1A1* mRNA expression was found to be decreased when compared to control animals [62]. This agrees with our eQTL results. There is also evidence that induction of *CYP1A1* is a risk factor for hypertension [63]. This in turn may explain the protective effect of the genetic variant rs2168518, which reduces the expression of *CYP1A1* on blood pressure. Further investigations on the impact of *CYP1A1* expression in cell lines with a defined genotype after oxidative stress induction could be an interesting field of future research.

Another interesting, although elusive finding in our study, is the gender-specificity of the rs2168518 association with AMD. There are three phenotypes colocalizing with the rs2168518-associated signal including AMD, blood pressure, and hearing difficulty/problems with background noise. While there is no gender-specific association with blood pressure, the AMD association is male-specific and the hearing difficulty association signal shows a female specificity. This supports a potential gender-specific regulation mechanism at this locus. However, our approach was not feasible to draw additional conclusions regarding gender-specific eQTL effects as the sample size and statistical power in the eQTL dataset was insufficient. Nevertheless, an association between the aforementioned polymorphism and certain blood pressure medications, which was calculated separately in females and males, colocalizes with different eGenes in each gender. While the association signal in females colocalizes mainly with *MPI* and *ULK3*, the association signal in males colocalizes predominantly with *CYP1A1* and *CSK*. This fits the colocalization of *CYP1A1* expression with the association signal of AMD and the fact that we could find an association only in male AMD patients. It should be noted, however, that so far this is purely speculative and requires further analyses. Nonetheless, alterations in the binding sites for the sex determination TFs SRY and SOX9 [43] as well as for the X chromosomal TF ZFX indicated that gender-specific regulation processes could be involved at this locus. As these aspects cannot be clarified conclusively at the present time, it is currently not recommended to introduce rs2168518 genotyping into clinical practice.

An in-depth characterization of a novel AMD-associated locus at 15q24.1 reveals several effects with potential impact on AMD pathogenesis. Importantly, we described an intriguing link between gene expression regulation of *CYP1A1* and CNV as well as blood pressure. Together these findings close the gap between functional evidence from previous studies and the genetics of AMD. Furthermore, several altered TF binding sites at 15q24.1 could contribute to gender specificity of the AMD association at this locus. Finally, our data emphasize the importance to identify promising candidate genes at an associated locus in order to reach a better insight into disease mechanisms behind a genetic association signal.

## Figures and Tables

**Table 1:** Association of rs2168518 with AMD subtypes in the IAMDGC dataset. For all associations, the A allele indicates the effect allele. Associations were calculated in comparison with the respective non-AMD individuals. Q-values < 0.05 are indicated in bold.

	Sample Size	OR [95% CI]	*p*-Value	Q-Value
Late-stage AMD	33,976	1.089 [1.052–1.127]	8.74 × 10^−7^	**4.00 × 10^−6^**
Geographic atrophy (GA)	21,067	1.077 [1.016–1.142]	0.013	**0.024**
Choroidal neovascularization (CNV)	**28,581**	1.091 [1.050–1.133]	8.29E-06	**1.87 × 10^−5^**
GA & CNV	19,992	1.073 [1.001–1.150]	0.048	0.054
Early stage AMD	24,489	1.036 [0.992–1.081]	0.112	0.112
<75 years	17,326	1.121 [1.066–1.177]	7.41 × 10^−6^	**1.87 × 10^−5^**
>75 years	15,825	1.055 [1.005–1.108]	0.030	**0.039**
Male	14,352	1.139 [1.081–1.200]	8.89 × 10^−7^	**4.00 × 10^−6^**
Female	19,624	1.053 [1.007–1.102]	0.025	**0.038**

OR = odds ratio, CI = confidence intervals, Q-value = false discovery rate (FDR)-corrected *p*-value.

**Table 2:** Haplotype distribution of genetic variants in LD (R^2^ > 0.8 in Europeans) with rs2168518 in different populations. Haplotype frequencies were calculated with the LDhap tool from LDlink [26,27].

Haplotype	rs2168518 Allele	EUR	EAS	SAS	AFR
**H1**	A	0.5577	0.1508	0.1483	0.0257
**H2**	G	0.3429	0.5149	0.7321	0.7973
**H3**	G	0.0268	0.2788	0.0828	0.0408
**H4**	A	0.0109	-	-	-
**H5**	A	-	0.0169	-	-
**H6**	G	-	-	-	0.0983
**H7**	G	-	-	-	0.0106

EUR = European population, EAS = East Asian population, SAS = South Asian population, AFR = African population.

**Table 3:** Association of rs2168518 in the UK Biobank genome-wide association studies (GWAS) summary statistics. Fifteen phenotypes display a significant association with rs2168518 (*p*-value < 1 × 10^−4^) in the UK Biobank PheWeb browser [28]. The association of rs2168518 with Macular Degeneration failed to reach the Table 19. Gender specificity (probability > 0.8 in one gender) was defined by comparison of the association signal in males and females in the UK Biobank summary statistic results using colocalization. Further, a colocalization of the respective signal with the late-stage AMD signal in the IAMDGC dataset (both sexes) was calculated. Coloc probabilities for “one signal” indicate that the same genetic signal underlies the association with AMD and the respective phenotype, while probabilities for “two signals” indicate that different genetic variants are underlying the associations. Genome-wide association *p*-values (< 5 × 10^−8^) are indicated in bold, as well as coloc probabilities > 0.8.

	PheWeb Association	Summary Statistics Association			Coloc Probability with IAMDGC AMD	
UK Biobank Phenotype	*p*-Value	Gender	*p*-Value	Gender Specificity	Same Signal	Two Signals
Diastolic blood pressure, automated reading	**1.50 × 10^−23^**	both sexes	**2.27 × 10^−27^**	no	0.604	0.385
		female	**7.27 × 10^−18^**		**0.964**	0.035
		male	**2.58 × 10^−11^**		0.610	0.379
Vascular/heart problems diagnosed by doctor: high blood pressure	**7.20 × 10^−23^**	both sexes	**2.24 × 10^−26^**	no	**0.976**	0.024
		female	**2.89 × 10^−14^**		**0.918**	0.079
		male	**9.90 × 10^−14^**		**0.970**	0.029
Vascular/heart problems diagnosed by doctor: none of the above	**2.60 × 10^−21^**	both sexes	**2.79 × 10^−25^**	no	**0.970**	0.029
		female	**9.10 × 10^−14^**		**0.860**	0.136
		male	**4.11 × 10^−13^**		**0.969**	0.030
Non-cancer illness code, self-reported: hypertension	**2.70 × 10^−21^**	both sexes	**2.15 × 10^−24^**	no	**0.975**	0.024
		female	**3.15 × 10^−13^**		**0.936**	0.062
		male	**9.12 × 10^−13^**		**0.965**	0.034
Systolic blood pressure, automated reading	**1.60 × 10^−11^**	both sexes	**9.33 × 10^−14^**	no	0.735	0.258
		female	**1.12 × 10^−10^**		**0.969**	0.030
		male	7.66 × 10^−5^		0.428	0.523
Medication for cholesterol, blood pressure or diabetes: blood pressure medication	**6.30 × 10^−11^**	male	**3.63 × 10^−12^**		**0.938**	0.061
Medication for cholesterol, blood pressure, diabetes, or take exogenous hormones: blood pressure medication	**3.90 × 10^−10^**	female	**8.58 × 10^−12^**		**0.890**	0.106
Creatinine (enzymatic) in urine	**5.30 × 10^−9^**	both sexes	2.85 × 10^−7^	no	0.037	**0.936**
		female	8.92 × 10^−8^		0.038	**0.934**
		male	0.031		0.037	**0.936**
Treatment/medication code: ramipril	7.40 × 10^−8^	both sexes	8.01 × 10^−8^	yes, only in male	**0.964**	0.035
		female	0.103		0.034	0.067
		male	**4.03 × 10^−8^**		**0.898**	0.099
Treatment/medication code: bendroflumethiazide	1.20 × 10^−7^	both sexes	5.29 × 10^−8^	no	**0.948**	0.051
		female	9.58 × 10^−6^		**0.901**	0.080
		male	1.37 × 10^−3^		0.486	0.085
Medication for cholesterol, blood pressure or diabetes: none of the above	1.30 × 10^−7^	male	**1.23 × 10^−8^**		**0.830**	0.165
Hearing difficulty/problems with background noise	4.10 × 10^−6^	both sexes	1.43 × 10^−6^	yes, only in female	**0.917**	0.079
		female	**6.01 × 10^−9^**		**0.966**	0.033
		male	0.345		0.007	0.062
Birth weight of first child	1.20 × 10^−5^	female	2.48 × 10^−5^		0.785	0.175
Mineral and other dietary supplements: glucosamine	2.50 × 10^−5^	both sexes	3.59 × 10^−5^	no	**0.877**	0.068
		female	9.41 × 10^−3^		0.118	0.098
		male	8.17 × 10^−4^		0.469	0.056
Sodium in urine	7.20 × 10^−5^	both sexes	8.38 × 10^−4^	no	0.037	**0.935**
		female	1.58 × 10^−3^		0.037	**0.935**
		male	0.106		0.010	0.300
Eye problems/disorders: macular degeneration	5.10 × 10^−3^	both sexes	2.66 × 10^−3^			
		female	0.157			
		male	2.82 × 10^−3^			

## Supplementary Materials

The following are available online at https://www.mdpi.com/2073-4409/9/10/2257/s1, Figure S1: Conditional analysis to delineate the AMD association signal at 15q24.1, Figure S2: Colocalization analysis of eGenes in adipose subcutaneous tissue, Table S1: Gender distribution in the investigated subgroups in the IAMDGC dataset, Table S2: Genetic variants in LD (R^2^ > 0.8) with rs2168518 in Europeans, Table S3: Haplotypes at 15q24.1, Table S4: Association of rs2168518 in the UK Biobank GWAS summary statistics, Table S5: EQTL analysis of rs2168518 in GTEx, Table S6: Predicted binding sites in eGenes for hsa-mir-4513, Table S7: Colocalization analysis of eGenes, Table S8: Colocalization analysis of eQTL results with phenotype associations from IAMDGC and UK Biobank, Table S9: Functional annotation of genetic variants in LD with rs2168518 (R^2^ > 0.8 in Europeans) with RegulomeDB 2.0.

## International AMD Genomics Consortium (IAMDGC)

Lars G. Fritsche, Wilmar Igl, Jessica N. Cooke Bailey, Felix Grassmann, Sebanti Sengupta, Jennifer L. Bragg-Gresham, Kathryn P. Burdon, Scott J. Hebbring, Cindy Wen, Mathias Gorski, Ivana K. Kim, David Cho, Donald Zack, Eric Souied, Hendrik P.N. Scholl, Elisa Bala, Kristine E. Lee, David J. Hunter, Rebecca J. Sardell, Paul Mitchell, Joanna E. Merriam, Valentina Cipriani, Joshua D. Hoffman, Tina Schick, Yara T.E. Lechanteur, Robyn H. Guymer, Matthew P. Johnson, Yingda Jiang, Chloe M. Stanton, Gabriëlle H.S. Buitendijk, Xiaowei Zhan, Alan M. Kwong, Alexis Boleda, Matthew Brooks, Linn Gieser, Rinki Ratnapriya, Kari E. Branham, Johanna R. Foerster, John R. Heckenlively, Mohammad I. Othman, Brendan J. Vote, Helena Hai Liang, Emmanuelle Souzeau, Ian L. McAllister, Timothy Isaacs, Janette Hall, Stewart Lake, David A. Mackey, Ian J. Constable, Jamie E. Craig, Terrie E. Kitchner, Zhenglin Yang, Zhiguang Su, Hongrong Luo, Daniel Chen, Hong Ouyang, Ken Flagg, Danni Lin, Guanping Mao, Henry Ferreyra, Klaus Stark, Claudia N. von Strachwitz, Armin Wolf, Caroline Brandl, Guenther Rudolph, Matthias Olden, Margaux A. Morrison, Denise J. Morgan, Matthew Schu, Jeeyun Ahn, Giuliana Silvestri, Evangelia E. Tsironi, Kyu Hyung Park, Lindsay A. Farrer, Anton Orlin, Alexander Brucker, Mingyao Li, Christine Curcio, Saddek Mohand-Saïd, José-Alain Sahel, Isabelle Audo, Mustapha Benchaboune, Angela J. Cree, Christina A. Rennie, Srinivas V. Goverdhan, Michelle Grunin, Shira Hagbi-Levi, Peter Campochiaro, Nicholas Katsanis, Frank G Holz, Frédéric Blond, Hélène Blanché, Jean-François Deleuze, Robert P. Igo Jr., Barbara Truitt, Neal S. Peachey, Stacy M. Meuer, Chelsea E. Myers, Emily L. Moore, Ronald Klein, Michael A. Hauser, Eric A. Postel, Monique D. Courtenay, Stephen G. Schwartz, Jaclyn L. Kovach, William K. Scott, Gerald Liew, Ava G. Tfan, Bamini Gopinath, John C. Merriam, R. Theodore Smith, Jane C. Khan, Humma Shahid, Anthony T. Moore, J. Allie McGrath, Reneé Laux, Milam A. Brantley Jr., Anita Agarwal, Lebriz Ersoy, Albert Caramoy, Thomas Langmann, Nicole T.M. Saksens, Eiko K. de Jong, Carel B. Hoyng, Melinda S. Cain, Andrea J. Richardson, Tammy M. Martin, John Blangero, Daniel E. Weeks, Bal Dhillon, Cornelia M. van Duijn, Kimberly F. Doheny, Jane Romm, Caroline C.W. Klaver, Caroline Hayward, Michael B. Gorin, Michael L. Klein, Paul N. Baird, Anneke I. den Hollander, Sascha Fauser, John R.W. Yates, Rando Allikmets, Jie Jin Wang, Debra A. Schaumberg, Barbara E.K. Klein, Stephanie A. Hagstrom, Itay Chowers, Andrew J. Lotery, Thierry Léveillard, Kang Zhang, Murray H. Brilliant, Alex W. Hewitt, Anand Swaroop, Emily Y. Chew, Margaret A. Pericak-Vance, Margaret DeAngelis, Dwight Stambolian, Jonathan L. Haines, Sudha K. Iyengar, Bernhard H.F. Weber, Gonçalo R. Abecasis, Iris M. Heid.
